# Pedf derived peptides affect colorectal cancer cell lines resistance and tumour re-growth capacity

**DOI:** 10.18632/oncotarget.26085

**Published:** 2019-04-26

**Authors:** Paloma Honrubia-Gómez, María-Pilar López-Garrido, Carmen Gil-Gas, José Sánchez-Sánchez, Carmen Alvarez-Simon, Jorge Cuenca-Escalona, Ana Ferrer Perez, Enrique Arias, Raul Moreno, Francisco Sánchez-Sánchez, Carmen Ramirez-Castillejo

**Affiliations:** ^1^ Stem Cell Laboratory, Departamento Ciencias Médicas, CRIB, UCLM, Albacete, Spain; ^2^ Genética Médica, Departamento de Ciencia y Tecnología Agroforestal y Genética, IDINE, UCLM, Albacete, Spain; ^3^ Current address: Unidad de Oncologia, Hospital de Almansa, Albacete, Spain; ^4^ Cancer Stem Cell Laboratory, HST Group, Biotechnology and V Biology Department, ETSIAAB, UPM, Madrid, Spain; ^5^ Current address: Oncology Division, Hospital Obispo Polanco, Teruel, Spain; ^6^ Departamento de Sistemas Informáticos, UCLM, Albacete, Spain; ^7^ UCAM, UCLM, Toledo, Spain

**Keywords:** colorectal cancer stem cells, cancer initiating cells, relapse, self-renewal inhibition, PEDF

## Abstract

Relapse after chemotherapy treatment depends on the cancer initiating cells (CICs). PEDF (Pigmented Epithelium Derived Factor) is an anti-angiogenic, neurotrophic and self-renewal regulator molecule, also involved in CICs biology. Acute and chronic exposition of colon cancer cell lines to CT/CTE PEDF-derived peptides decreased drug-resistance to conventional colorectal cancer treatments, such as oxaliplatin or irinotecan. We confirmed a reduction in the irinotecan and oxaliplatin IC50 doses for all tested tumour cell lines. After xenograft transplantation, CT/CTE treatments also produced a reduction in resistance to conventional chemotherapy treatments as in culture-assays. Metastatic capacity of these treated cell lines was also depleted. The PEDF signaling pathway could be a future therapeutic tool for use as an adjuvant therapy that decreases IC50 dosis, adverse effects and treatment costs. This pathway could also be involved in an increase of the time relapse in patients, decreased tumourigenicity, and decreased capacity to produce metastasis.

## INTRODUCTION

There are different hypotheses about the origin of tumours, none of which has yet been able to explain all cases. One such hypothesis is based on the high susceptibility of stem cells to accumulate mutations, together with the existence of epigenetic changes that are essential for the initiation and development of tumourgenic behaviour. For this reason tumour stem cells display some same general characteristics as healthy stem cells (SCs) such as quiescence, self-renewal, asymmetric cell division, cell regulation via Wnt, Notch, Sonic Hedgehog signaling (Shh), and resistance to toxic substances and drugs. All of these characteristics were postulated more than fifteen years ago [1–4]. Therefore stem cells undergo long cell cycle rates in concert with maintenance of the quiescent state. In this way, stem cells could turn into tumour stem cells. According to this hypothesis, tumours would be complex cell-subsets, even nameable as tissues, with the growth capacity and disorganized framework of the tumour being triggered by a small subset of tumour pathological cells exhibiting the characteristics of stem cells. This could also be the case in colorectal cancer [5].

Self-renewal can be defined as the capacity of the cell to, at a minimum, originate a daughter cell with identical characteristics to the previous one. Consequently, stem cells can undergo multiple cell divisions maintaining their cell de-differentiation. Self-renewal is also produced by asymmetrical cell divisions of stem cells. This ability is essential for maintaining stem cells throughout the organism′s lifetime. Every stem cell must maintain an equilibrium between self- renewal and cell differentiation [1].

PEDF (Pigment Epithelium-Derived Factor) protein has been related to the modulation of self-renewal mechanisms in stem cells [6, 7]. PEDF protein belongs to the serpin protein family, described as inhibitors of serin-proteases [8]. Nevertheless, PEDF does not have this function [9] and is known to stimulate self-renewal in stem cells, with no effect on the system’s proliferation. This stimulus could be competitively reduced by the C-terminal (CT) fragment of the protein. Consequently there would be a depletion in the amount of cells present in a culture [6].

PEDF exhibits various collaborative effects against tumour progression. PEDF may induce cellular differentiation and induce apoptosis in tumour cells [10–13]. Also, PEDF protein seems to be able to inhibit tumour cell proliferation, vascularization, cell migration, invasion, and metastasis. Accordingly, the PEDF protein can be described as a potent anti-neoplasic agent [14–16]. However, PEDF′s stemness capacity could be involved in cancer initiating cells’ self-renewal, as has been postulated by several research groups [17–19]. Here, we report on the use of the C-terminal domain of the PEDF protein as a tool to create competition with the native PEDF protein′s effect over TICs’ (tumor initiating cells) self-renewal capacity [6, 20]. We present the impact on tumour growth, as well as the backspin of this competition over chemotherapy resistance and self-renewal, or the relapse capacity of tumour-initiating cells in colorectal cancer cell lines.

## RESULTS

Here we present the *in vivo* and *in vitro* effects of the C-terminal part of PEDF on tumoural cell lines’ growth and tumourogenicity. We have measured the IC50 of different chemotherapeutic treatments in combination with two fragments of the PEDF protein: the CT and CTE peptides (these are identical peptides from the C-terminal part of the PEDF protein, differentiated by presence of a serine or glutamic residue). Also, we have measured the resistant population at the end of the treatment, which is an important date for comparing the effectivity of those treatments. We have used three different colorectal cancer cell lines, with different genetic expression hallmarks (SW-480, SW-620 and DLD-1). A fourth cell line, HT29 was also used for comparison in some of the experiments due to the high oncogenicity of those cells. PEDF derived peptides have been used in combined treatment with conventional chemotherapy, oxaliplatin and irinotecan, both in first and second line chemotherapy for colorectal cancer patients. These two PEDF derived peptides are designed from the carboxi-terminal part of the PEDF protein. CTE is the same molecule as CT, from the C-terminal part of PEDF protein, but with a glutamic acid instead of the phosphorylable serine of this small molecule. The treatments employed in this paper were acute treatments, lasting two hours, and chronic treatments, lasting 6 weeks, with the cell culture medium. In both cases the optimum concentration was 8 nM, optimized in a previous work-group in murine neural models [6] and in a colon cancer cell line, SW-480 (Supplementary Figure 1).

We have selected three different colorectal cancer cell lines in order to study the generic effect in multiple source colorectal tumours, and the chemotherapy used will be oxaliplatin and irinotecan, which are the most common first line chemotherapy agents used for colorectal cancer patients.

### Decrease of chemotherapy resistance

The resistance to chemotherapy decreased in the cell lines (DLD-1, SW-480 and SW-620) treated with PEDF derived peptides (CT and CTE). All the cell lines showed statistically significant reduction of IC50, oscillating between 20 and 70% depending on every cell line in both acute and chronic treatments.

**Figure 1:**
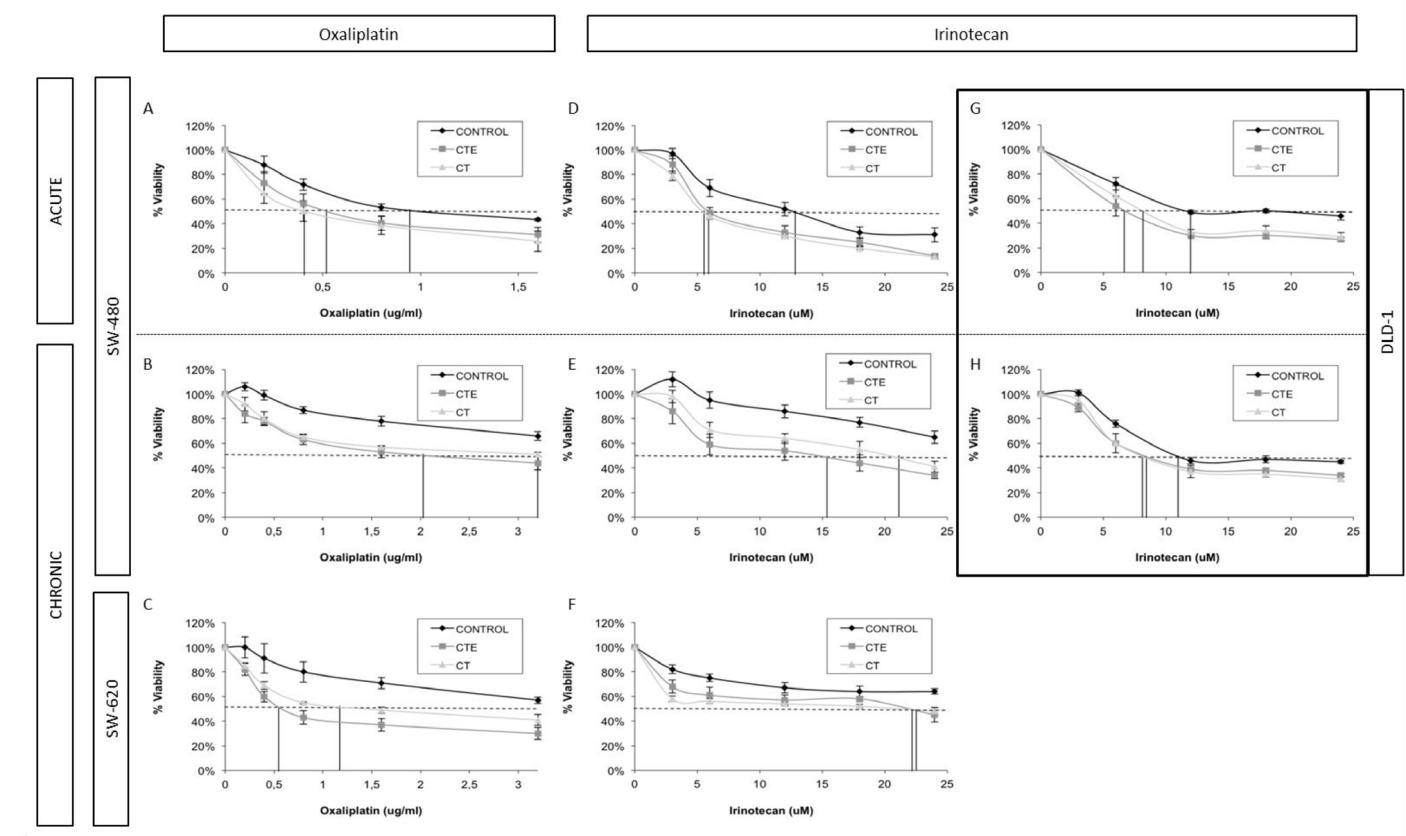
Changes in IC_50_ and doses-response curve behaviour in different colorectal cancer cell lines with different chemotherapeutic treatments, after ct and cte peptides in acute and chronic treatment (**A**) Oxaliplatin dose-response curves of SW-480 cell line with or without CT and CTE acute treatment. (**B**) Oxaliplatin dose-response curves of SW-480 cell line with or without CT and CTE chronic treatment. (**C**) Oxaliplatin dose-response curves of SW-620 cell line with or without CT and CTE chronic treatment. (**D**) Irinotecan dose-response curves of SW-480 cell line with or without CT and CTE acute treatment. (**E**) Irinotecan dose-response curves of SW-480 cell line with or without CT and CTE chronic treatment. (**F**) Irinotecan dose-response curves of SW-620 cell line with or without CT and CTE chronic treatment. (**G**) Irinotecan dose-response curves of DLD-1 cell line with or without CT and CTE acute treatment. (**H**) Irinotecan dose-response curves of DLD-1 cell line with or without CT and CTE chronic treatment. Data represented as mean ± SEM.

SW-480 and SW-620 cell lines showed a significant reduction, between 30 to 50% in the variables studied: IC50 and resistant population. All these parameters and the survival curves with PEDF-derived peptides are always under control survival curves (Figure 1A–1F).

In the SW-480 cell line there is a sharp 50% decrease of oxaliplatin and irinotecan IC50 value when they are combined with CT and CTE chronic or acute treatments (Table 1). A reduction in the final resistant-cell percentage has also been observed in these assays, with oxaliplatin and irinotecan combined with CT or CTE treatments. We observed a stark 30–50% decrease for acute and chronic treatments of less resistant-cell population (Table 2).

**Table 1:** Oxaliplatin and irinotecan IC50 in monotreatment (first column) and with CT (second column) and CTE (third column) PEDF derived peptides treatment in acute and chronic administration

ug/ml	Cell lines	Oxaliplatin	+CT	+CTE
Acute treatment	**SW-480**	1.4 ± 0.2	0.7 ± 0.3 (50%)^**^	1.1 ± 0.2 (24, 4%)^*^
	**SW-620**	1.4 ± 0.6	1.3 ± 0.9	1.0 ± 0.2
	**DLD-1**	8.8 ± 0.9	7.0 ± 0.4	8.3 ± 0.6
	**HT-29**	5.4 ± 0.3	3.1 ± 0.1^*^ (42.6%)	3.4 ± 0.2^*^(37%)
Chronic treatment	**SW-480**	9.0 ± 1.4	4.0 ± 0.6^*^ (55.6%)	2.7 ± 0.8^*^ (70%)
	**SW-620**	6.3 ± 1.0	2.9 ± 0.8^*^ (54%)	1.5 ± 0.3^*^ (76.2%)
	**DLD-1**	12.3 ± 0.5	10.7 ± 0.5	13.1 ± 0.9
	**HT-29**	5.7 ± 0.1	5.4 ± 0.7	5.1 ± 0.1^*^ (10.5%)

ug/ml	Cell lines	Irinotecan	+CT	+CTE
Acute treatment	**SW-480**	14.4 ± 2.4	9.3 ± 0.3^*^ (35.4%)	9.7 ± 0.5^*^ (32.6%)
	**SW-620**	63 ± 2	33.8 ± 0.9 (46%)	41 ±2 (35%)
	**DLD-1**	10.9 ± 0.5	8.5 ± 0.6^*^ (22%)	7.8 ± 0.5^*^ (28.4%)
	**HT-29**	13.7 ± 0.2	13.3 ± 0.4	11.9 ± 0.1^*^ (13%)
Chronic treatment	**SW-480**	42.7 ± 0.2	25.3 ± 0.9^*^ (40.7%)	18.3 ± 0.2^*^ (57%)
	**SW-620**	30 ± 3	18 ± 1^*^ (40%)	20 ± 1^*^ (33.3%)
	**DLD-1**	11.2 ± 0.5	8.9 ± 0.6^*^ (20.5%)	9.9 ± 0.4^*^ (11.6%)
	**HT-29**	11.2 ± 0.2	11.1 ± 0.2	9.4 ± 0.1^*^ (16%)

The IC_50_ percentage decrease in the combined treatments is shown in brackets. IC50 concentrations are expresssed in ug/ml. ^*^p < 0.5; ^**^p < 0.5 and *n* ≥ 3 for every condition.

**Table 2:** Percentage of resistant cells after oxaliplatin and irinotecan treatments combined with CT and CTE PEDF peptides in acute and chronic administration

ug/ml	Cell lines	Oxaliplatin	+CT	+CTE
Acute treatment	**SW-480**	43 ± 2	26 ± 9 (39, 5%)^*^	31 ± 6 (28%)^*^
	**SW-620**	50 ± 1	31 ± 5 (38%) ^*^	31 ± 3 (38%)^*^
	**DLD-1**	33 ± 5	28 ± 5 (15%)	30 ± 3 (9%)
	**HT-29**	43 ± 2	33.4 ± 0.5 (22%)^*^	36.5 ± 0.5 (15%)^*^
Chronic treatment	**SW-480**	66 ± 4	51 ± 2 (23%)^*^	47 ± 5 (29%)^*^
	**SW-620**	57 ± 2	41 ± 5 (28%)^*^	33 ± 6 (42%)^*^
	**DLD-1**	33 ± 6	29 ± (12%)	31 ± 1 (6%)
	**HT-29**	44 ± 2	35 ± 3 (21%)	34 ± 3 (23%)

ug/ml	Cell lines	Irinotecan	+CT	+CTE
Acute treatment	**SW-480**	31 ± 7	14 ± 3 (55%)^*^	15 ± 2 (52%)^*^
	**SW-620**	65 ± 5	41 ± 5 (37%)^*^	34 ± 2 (48%)^*^
	**DLD-1**	46 ± 4	29 ± 2 (37%)^**^	34 ± 1 (26%)^*^
	**HT-29**	21 ± 3	16 ± 5 (24%)^*^	17.2 ± 0.8 (18%)^*^
Chronic treatment	**SW-480**	65 ± 5	41 ± 5 (37%)^*^	34 ± 3 (48%)^*^
	**SW-620**	64 ± 2	48 ± 3 (25%)^*^	44 ± 6 (31%)^*^
	**DLD-1**	45 ± 1	31 ± 2 (31%)^*^	34 ± 2 (24%)^*^
	**HT-29**	22 ± 3	20 ± 2 (9%)	14 ± 1 (36%)^*^

The percentage decrease of resistant-cells after the combined treatment with chemotherapy plus one of the PEDF derived peptides is shown in brackets. ^*^p < 0.5; ^**^p < 0.5 and *n* ≥ 3 for every condition.

As previously mentioned, similar data was reported for the SW-620 cell line (Figure 1A–1F). We observed a statistically significant decrease in the IC50 of oxaliplatin, at least 30% with acute and 50–70% wtih chronic treatments. For irinotecan, there is also an important IC50 decrease of 35% with chronic treatments of CT and CTE PEDF derived peptides (at least *p* < 0.5 and *n* ≥ 3 for every condition) (Table 1). This decreasing tendency of IC50 is also observable in acute treatments, but without statistically significant differences (Table 1) (Supplementary Figure 2). Resistant population in these assays was reduced by 40–30% in acute and chronic treatments with oxaliplatin and CT or CTE, and 40–50% with irinotecan (Table 2).

After the dose-response assays, we verified that the DLD-1 cell line is more sensitive to irinotecan than oxaliplatin. The results showed an important IC50 decrease with both treatments CT and CTE peptides (Figure 1G–1H). The IC50 of the combined treatment is slightly less than that of oxaliplatin alone and showed a statistically significant 20–30% decrease compared to irinotecan treatment (at least *p* < 0.5 and *n* ≥ 3 for every condition) (Table 1). In this assay, the IC0 (inferred data dose needed to eliminate the total resistant population), in both oxaliplatin and irinotecan treatments, showed a reduction (Supplementary Figure 3). Resistant population was especially reduced with the irinotecan combination treatment, by 20–30% for acute and chronic treatments respectively (Table 2).

HT-29 also showed a decrease in the IC50 of the chemotherapy combined with PEDF-derived peptides. A significant response was observed in acute CT/CTE combined with oxaliplatin treatment, obtaining a decrease of 40% in the oxaliplatin IC50. Reduction of IC0 was also visible (Supplementary Figure 4) and the resistant population suffered a decrease of around 20% with both oxaliplatin and irinotecan (Table 2). Chronic treatments revealed similar results (Table 1), 10% IC50 oxaliplatin reduction and 15% with irinotecan. Resistant population in these assays was reduced by 20% with oxaliplatin and CT or CTE, and up to 35% of cells with irinotecan (Table 2).

### The tumourigenicity *in vivo* decreases with pedf derived-peptides treatments

*In vivo* xenograft assays were started with the injection of 500 and 5,000 tumoral cells in nude mice to follow the tumour growth in the mice flank. Those cells were previously exposed to chronic treatment with CT and CTE peptides during six passages/2 weeks. Controls are xenografts with the same number of untreated injected cells. The assays were performed in parallel with the three colorectal cell lines. In this type of assays we compared the growth of the tumour in the flank between control untreated cells and CT or CTE previously treated cells.

*In vivo* assays showed a significant decrease in the tumourigenicity of tumour cells after treatment with PEDF derived peptides in DLD-1, SW-480 and SW-620 cells lines. Consequently, the observed behaviour varied depending on the origin and the molecular characteristics of every cell line. CT treatment showed less effectivity than CTE-PEDF *in vivo*. CTE peptide is more stable due to the negative charge in its glutamic acid (instead of the serine found in the CT peptide).

**Figure 2:**
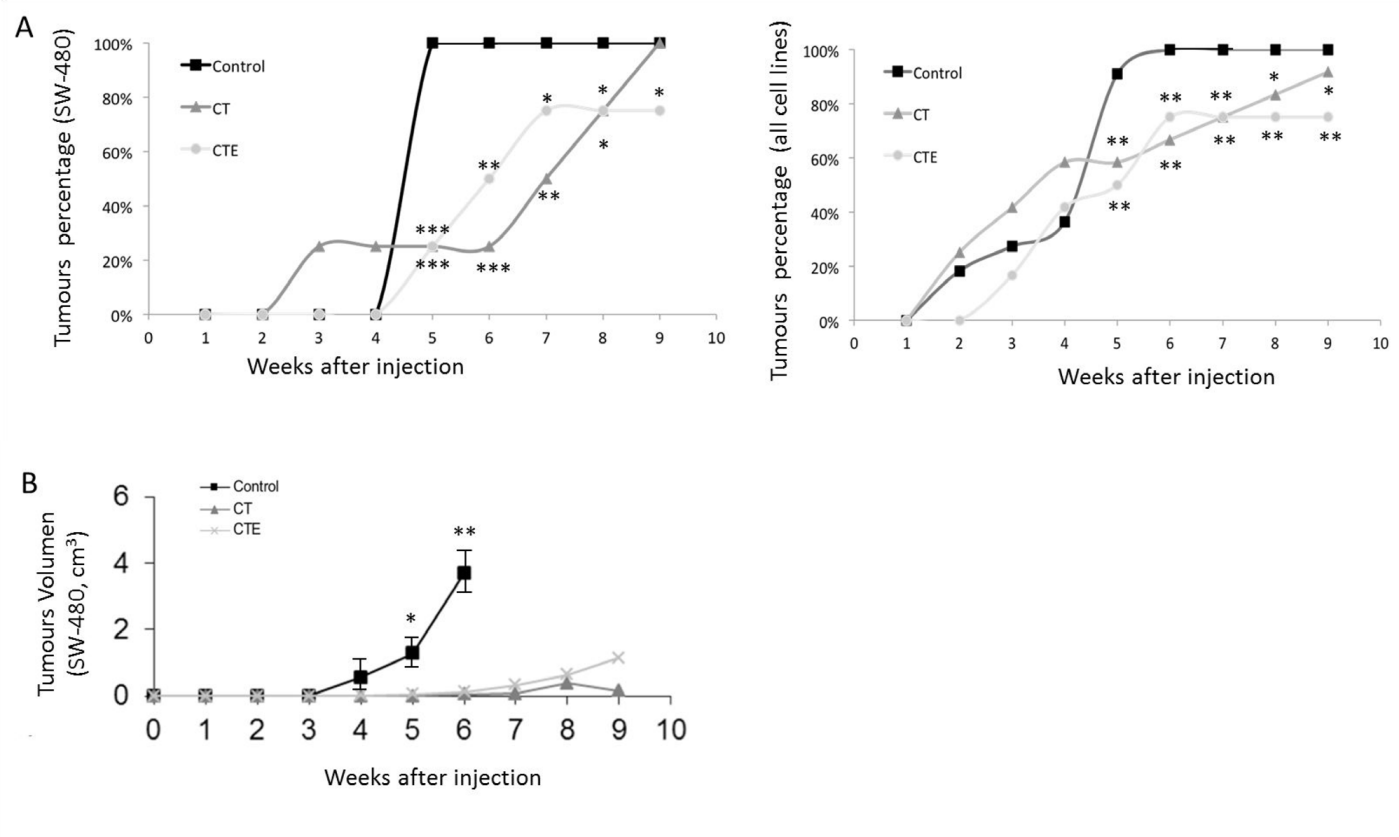
Timing evolution of size and tumours number after xenograft injection, with or without CT and CTE treatments (**A**) Number of xenograft tumours decrease after CT and CTE treatments in SW-480 cell line model (left graph). Twelve mice were injected, four animals for every experimental condition. In the right graph, the total number of the tumours formed from the total colon cell lines used has been represented. The same effect as in the SW-480 xenograft was observed and the total number of injected mice was 35, three to four animals for every experimental condition (right graph). (**B**) Progression of the xenograft size (in cm^3^) with or without CT and CTE treatments in SW-480 cell line model. Data represented as mean ± SEM (^*^p > 0.05, ^**^p > 0.01, ^***^p > 0.001).

Notably, SW-480 cell line assays confirmed a 25% reduction in the number of tumours after CTE treatment, and similar results with CT treatment (Figure 2A, to the left). As has been represented in Figure 2A′s left graph, four weeks after xenograft injection, when 100% of the tumours with untreated cells are clearly visible, only 25% of the tumours are appreciable in treated xenografts. The total number of injected mice was twelve, four for every treatment. Furthermore, there is a clear delay in the appearance of the rest of the tumours after treatments with CT and CTE. The CT/CTE treatment caused a significant lag in the development of tumours, from four to eight weeks in the entire cell lines that were analysed (Figure 2A right).

SW-620 and DLD-1 cell lines exposed to PEDF-derived peptides in chronic treatment, resulted in a reduction of the developed tumour number in the same way as SW-480. We observed 50% fewer tumours as compared to the untreated cells.

When taken together, the colorectal cell lines results from injections within 35 mice showed that the number of tumours was significantly lower after treatment than of the control tumours. Comparing the data of all tumour lines used, 90% of the tumours of untreated cells are observed at four weeks and only 50% of the treated xenografts developed tumours.

Concerning the volume of the tumours, untreated SW-480 cells reached four times larger tumour sizes after four weeks, than those treated did in eight weeks. After 6 weeks, treated tumours were 94% smaller in size than untreated control ones. In addition, the beginning of the tumours was drastically delayed in treated cells when compared to the control’s: In the untreated control group, 90% of tumours appeared in the fourth week, while in treated groups this percentage was not reached until the eighth week (Figure 2B). Similar results are observed in the rest of the cell lines xenograft, with differences depending on the cell line′s growing rate.

### Pedf-derived peptides induced a significant decrease in chemotherapy resistance

**Figure 3:**
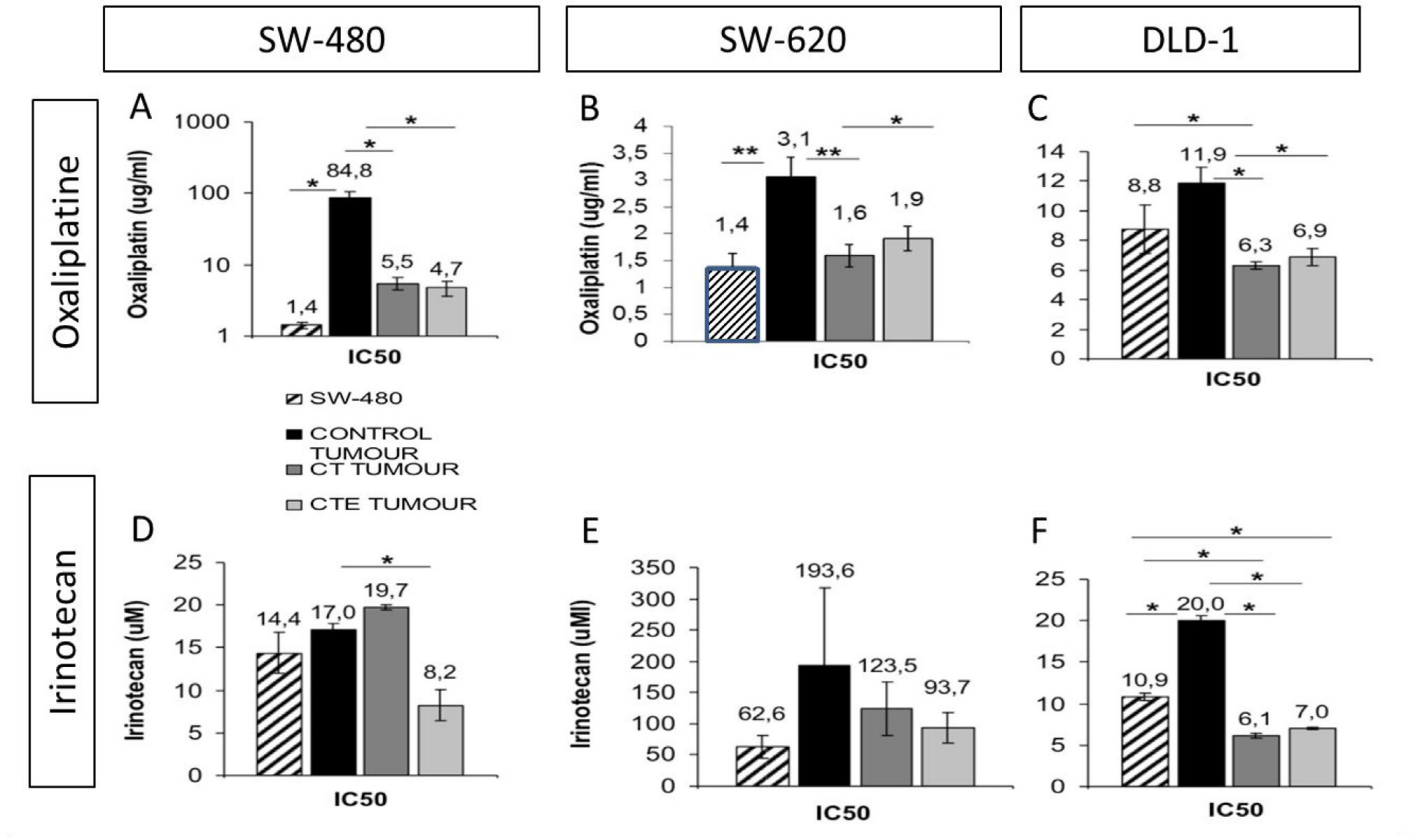
Oxaliplatin and irinotecan IC_50_ decrease after CT and CTE treatments in xenograft tumoral cells in culture Cell line (chart graDed column) is always less resistant than tumoral cells from the xenograft (chart solid columns). (**A**–**C**) Oxaliplatin IC50 with or without CT or CTE treatment, in xenograft from different colorectal cancer cell lines, A: SW-480, B: SW.620 and C: DLD-1. (**D**–**F**) Irinotecan IC50 with or without CT or CTE treatment, in xenograft from different colorectal cancer cell lines, A: SW-480, B: SW.620 and C: DLD-1. Data represented as mean ± SEM (^*^p > 0.05, ^**^p > 0.01, ^***^p > 0.001).

Tumour cells from xenografts were extracted, disgregated and re-cultivated in culture medium for a new chemotherapy treatment to study tumourigenicity and relapse capacity. We observed that cells from xenografts were more resistant to oxaliplatin and irinotecan chemotherapy than the original cell line. However, after cell treatments with CT or CTE peptides, treated xenograft-cells were less resistant (light and dark gray bars, in Figure 3) than untreated xenograft-cells (bars black, in Figure 3) and also less resistant than the original tumoural cell lines (bars white, in Figure 3). Treated cells responded to chemotherapy with a lower IC50 value for oxaliplatin and irinotecan than control untreated CT or CTE cells (Figure 3). Specifically, SW-480, the cell line that showed a decrease of around 50% in the tumour re-xenograft appearance after treatments, also produced a significant 25% decrease in the IC50 value of both CT and CTE treatments. This reduction is even more obvious with the oxaliplatin treatment, where the treatment does not reach IC50 in a physiological range, but treated xenograft cells had a huge reduction of above 90% of the IC50 with CT and CTE treatment. For irinotecan, only CTE treatment, but not CT, produced a 50% decrease in the IC50, *p* < 0.05 and *n* ≥ 3 every tumour groups (Table 3).

**Table 3:** Oxaliplatin and irinotecan IC50 dose after CT and CTE chronic treatment, in xenografted cells

ug/ml	Cell lines	Oxaliplatin	+ CT
Oxaliplatin treatment	**SW-480**	84,81 ± 21,92	5,51 ± 1,02 (93,50%)^*^
	**SW-620**	3,06 ± 0,36	1,59 ± 0,21 (48,04%)^**^
	**DLD-1**	11,87 ± 1,09	6,32 ± 0,23 (46,76%)^*^

ug/ml	Cell lines	Irinotecan	+ CT
Irinotecan treatment	**SW-480**	17,05 ± 0,76	19,7 ± 0,31
	**SW-620**	193,6 ± 30	123,5 ± 42,86 (36,21%)
	**DLD-1**	20,02 ± 0,61	6,11 ± 0,32 (69,48%)^*^

In brackets we show the reduction percentage of IC50.

Regarding SW-620 xenograft-cells, treatments produced a reduction in the IC50 with oxaliplatin of around 40% with CT and CTE treatment respectively, *p* < 0.01 and *p* < 0.05 and *n* ≥ 2 every tumour group. Irinotecan treatment also produced a 35–50% IC50 reduction, but the differences were not statistically significant, most likely because of the decrease in tumour occurence after treatments within these assays, and because the majority of the mice injected with treated cells did not produce any tumour.

### Pedf derived-peptides produce a decrease of *in vivo* relapse capacity (metastasis)

**Figure 4:**
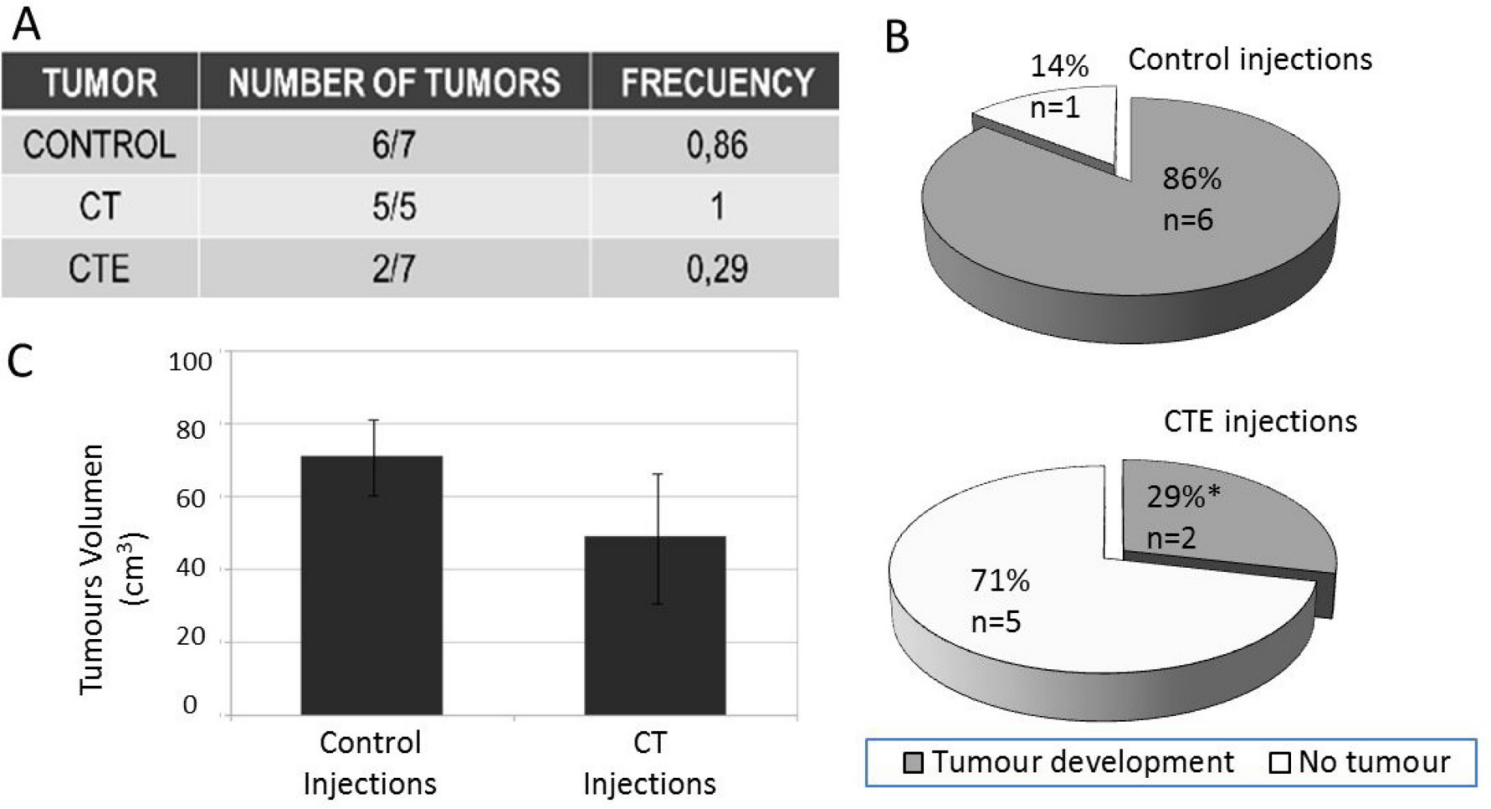
Re-xenograft after injections of treated xenograft cells treated with or without ct or cte peptides Re-xenograft represents the metastatic model, since they are secondary tumours from the injection of primary xenograft cells. SW-480 was used as a model in those Xenograft assys. (**A**) Frequency table with the total number of re-xenograft that have grown after CT or CTE treatments. (**B**) 360-degree graph that represents the tumour appearance frequency with or without CTE treatment. (**C**) CT treatment produced an identical tumour number but with 30% smaller size than the untreated re-xenograft injections. Diferenes are visual but not stadisticaly significant because *p* = 0.50174. Data represented as mean ± SEM.

Relapse capacity assays were designed using re-xenografts from different treatments and cultured *in vitro* after tumour dissociation. These cultured cells from the different conditions (Table 4), with and without exposition to PEDF-derived peptides, were re-injected in nude mice to study the capacity of those cells to again produce a new tumour. In our experiments, development of a new tumour occurred in less than 30% of cases (two out of seven CTE re-injected treated cells formed new tumours). Nineteen injections were performed in nude mice (Table in Figure 4A). From seven control mice with re-injected cells, six of them developed a tumour, whilst in the case of CTE treated cells, from seven re-injections just two (less than 30%) produced a new tumour (Figure 4B). Regarding the same experiment with CT treatment, all five injected mice developed tumours (Figure 4A). Nevertheless, tumour volumes of these re-xenografts were 30% smaller than control ones (Figure 4C).

**Table 4:** Percentage of resistant cells after oxaliplatin and irinotecan treatments combined with CT and CTE PEDF chronic administration in xenograft provenient cells

ug/ml	Cell lines	Control %	CT%	CTE%
Oxaliplatin	**DLD-1**	45,27 ± 2,97	34,95 ± 1,49 (22,80%)^*^	33,65 ± 3,94 (25,67%)^*^
	**SW-480**	59,39 ± 0,59	47,76 ± 0,48 (19,58%)	56,93 ± 10,07 (19,14%)
	**SW-620**	45,61 ± 1,85	44,01 ± 6,04 (3,51%)	36,88 ± 1,87 (19,14%)^*^

ug/ml	Cell lines	Control %	CT%	CTE%
Irinotecan	**DLD-1**	47,34 ± 3,64	38,71 ± 1,82 (18,23%)	37,41 ± 0,58 (20,98%)^*^
	**SW-480**	34,26 ± 3,80	39,2 ± 3,76	8,39 ± 3,27 (75,51%)^*^
	**SW-620**	29,25 ± 1,52	43,76 ± 8,90	52,09 ± 1,59^*^

In brackets is shown the percentage decrese of resistant-cells after the combined treatment with chemotherapy plus one of the PEDF derived peptides. ^*^p < 0.5 and *n* ≥ 2 for every condition.

## DISCUSSION

The main objective of this work is to show the effect of PEDF-derived peptides when they are combined with chemotherapeutic agents in clinical use. These peptides produce a significant decrease in the IC50 of irinotecan and oxaliplatin, which is decreased to 50% and even 70% for DLD-1, SW488 and SW-620 cell lines. This effect is observed in chronic but also in acute treatments, even at concentrations of 8 nM of PEDF-derived peptides, indicating a high enhancement effect. However, an inverse effect is observed between the aggressiveness of the tumour line and the possibility of counteracting the effect of resistance to chemotherapy. Therefore, early detection and avoidance of risk factors for the generation of resistances due to exposure remain of vital importance [21–24].

The neurotrophic and anti-angiogenic multifaceted factor PEDF [25–28] has three receptors capable of recognizing the native protein [29–32], however little is known about the molecular mechanisms that might be involved in the carboxil-end (CT) recognition. Regarding its molecular function, previous data demonstrated that when increasing CT dosis treatment in combination with exogenous native PEDF protein, CT showed competitive inhibitory behaviour on neural stem cells [33].

### Acute/chronic treatment approach

In order to study the PEDF protein′s stability, molecular functions, and the potential receptors involved in PEDF recognition, two different treatment approaches were carried out. The first one, an acute treatment, consisted of a single dose injection of 200 ng/ml (8 nM) that corresponds temporally with chemotherapy doses application, even oxaliplatin or irinotecan. This way, we investigated the mechanisms involved in the interaction between the chemotherapeutic compounds and the PEDF derived peptides. The second one, a chronic 6 week treatment, with 8 nM CT and CTE peptides, was carried out to study the effect of TICs self-renewal inhibition in metastatic transformation.

The peptide concentration (8 nM) was selected based on the team’s previous experience with the effect of this peptide on stem cell populations [6, 7]. An assay was also carried out in colorectal cancer cells to ensure that in that type of cells, the effect was observed in the same concentration ranges as in the previous studies used as references. The protective effect of PEDF in other model systems has been reported between 1 µg/ml [34] to 50 ng/ml [35], and that was the range tested in our assays. Supplementary data shows (Supplementary Figure 5) after a dose-response combined assay with oxaliplatin that the effect of the PEDF signalling pathway is observed in the same concentration range. An asymptotic effect is reached from 50 ng/ml to 800 ng/ml concentration, and we have selected an intermediate concentration, 200 ng/ml (8 nM) to prevent low effects being undetectable when using limited peptide concentrations. Higher concentrations have not been considered necessary because of the observed asymptotic effect, but could be tested in the future to improve the effect at the level of precision medicine.

Regarding the acute treatment approach, we observed a decrease from 50% to 75% in the resistance to chemotherapy. This decrease was observed in the four studied cell lines and with both chemotherapeutic agents, irinotecan and oxaliplatin. Even though acute treatments demonstrated lower efficacy than the chronic ones, the improvement of the acute treatment effects remains an essential task concerning possible future applications. In this sense, we have obtained important IC50 reductions in some of the treatments and cell lines. This promising fact would present potential advantages such as easier application, a less tedious application procedure for patients, and would be economically advantageous for the health system [22, 24]. In order to reach an efficient effect of the acute treatment based on the peptide concentration, distinct peptide concentration assays would have to be carried out. New dose assays with different amounts of PEDF-derived peptides might provide new clues for more effective removal treatments for the final resistant cell population.

Additionally, we observed a great improvement in the chronic treatments: tumour cell lines that were previously treated for 6 weeks with a constant 8 nM amount of CT and CTE peptides. The treatment application time was coordinated with the day of the cells passing process to obtain a more homogeneous response. During this assay, the main objective was to identify the role that these PEDF-derived peptides might be playing as regards cell division or signaling pathway activation related to stress induced by toxic substances [12, 20, 36]. After those chronic treatments were applied, over 80% of the chronic treatments exhibited a resistant decrease to chemotherapy treatments. Also we observed that different cell lines displayed a statistically significant reduction in the ability to develop tumours. Interestingly, in 100% of these tumours developed after chronic treatment, tumour size and re-xenograft capacity experimented a statistically significant depletion after chronic treatment. This effect could be related to the signaling pathway of the receptors activated by PEDF and their derived peptides employed here. There are some evidences of tumour-suppresors that inhibit epithelial mesenchymal transition (EMT), such as AIP1, which, working through the inhibition of VEGF-dependent signaling in the tumour niche, limit tumour growth and metastasis [37]. PEDF and VEGF are inter-regulated signals and could be one of the molecular bases of this potent therapeutic effect.

### Effect of the PEDF carboxil-end in the resistance to oxaliplatin and irinotecan

Here, we report the decreased resistance to chemotherapeutic agents, oxaliplatin and irinotecan, in four colorectal cancer cell lines when such chemotherapy is combined with a dose of PEDF-derived peptides (CT or CTE). Interestingly, this resistance varied depending on the chemotherapeutic agent used. Thus, we observed a major synergy between irinotecan and PEDF-derived peptides. This fact is possibly due to differences in the action mechanism of both drugs. As chemotherapeutics, oxaliplatin and irinotecan are cytotoxic compounds that affect cell division. In this sense, both irinotecan and oxaliplatin target different cellular mechanisms [38]. Therefore, the molecular function of irinotecan seems likely to justify the greater empowerment grade observed with PEDF-derived peptides over irinotecan than with these peptides and oxaliplatin [32, 33]. An adjustment in the doses and application timing of PEDF-derived peptides would be necessary in order to reach greater effectiveness of the treatment, surpassing the already promising results presented here, taking into account the physiological role of the PEDF signaling pathway in undifferentiated cells in different physiological niches [39, 40].

Considering the effect between PEDF peptides and the chemotherapeutic molecules used in this study, it is not possible to speak about synergism. We have tried to calculate the Combination Index to show if it was CI = 1 (additive effect) CI > 1 (antagonism) or CI < 1 (synergy). We have also performed a bibliographical study and concluded that the best methodology for this is based on the Median-Effect Equation (Chou) and the Combination Index Theorem (Chou-Talalay) (T.-C. Chou, 2010), [41] and the software for Drug Combination Computer Simulation: CompuSyn and Combenefit software that are availables for those calculations, as we describe in material and methods section. However, the data did not give us coherent results, which in our case could be because we cannot talk about synergy but about improvement, which does not have the same meaning. As Ting-Chao Chou points out: “synergism (or antagonism) is ‘mutual’, while improvement, enhancement or increase is ‘unilateral’” (Ting-Chao Chou, 2007, 2008), [42, 43]. Synergism or antagonism needs to be determined with IC values, while for potentiation, up or down regulation, we simply have to establish the x% enhancement or the Y-fold improvement. In our case, CT and CTE treatments, in and of themselves, have no effect (T.-C. Chou, 2010), so we cannot calculate Dm or m, both being values that are needed to calculate CI. The effect of these peptides is observed in combination with chemotherapy, so we see it as an enhancement of the effect of the chemotherapy, according to the definition itself. We consider the clarification of this important pharmacological-dynamic aspect relevant in order to know the pharmacological behaviour of those new molecules.

### Effect of the PEDF carboxil-end over tumours development

*In vivo* tumourigenicity of colorectal cell lines decreased with the application of a dose of PEDF-derived peptides. According to the previously presented results, PEDF-derived peptides, CT and CTE, showed to be notably effective against one of the most aggressive colorectal tumour cell lines, SW480, and also against their metastatic SW620 cell line, both derived from the same patient. In this sense, we observed the effect on the chemotherapy combined treatment, showing a decrease in the resistance of cells derived from xenografts previously treated with CT/CTE (light and dark gray bars in Figure 3), compared to cells from untreated xenografts (bars black in Figure 3). Not only the IC50 dose but also the final percentage of the resistant cell population after treatment with CT or CTE was observed.

The effect on the capacity for tumour development was also studied. Regarding the SW480 tumour cell line, we observed a 25% reduction of its capacity to develop new tumours in a xenograft model after CTE treatment. Notably, in SW620, the SW480-derived metastatic cell population, [44] we observed a 25% to 50% decrease in its capacity to develop new tumours in a xenograft model after CT or CTE treatment, respectively. The effect on the metastatic cell line SW620 could be explained by the fact that this tumour cell line has an enhanced aggressiveness grade, with a higher cell division kinetic, and therefore the peptides treatment effect generates a greater outcome than expected in basal conditions. It has been reported that therapeutic response to some treatments could be a function of the tumour metabolic phenotype [37].

### PEDF derived-peptides produce a decrease of *in vivo* relapse capacity (metastasis)

The difficulty of finding an experimental model of the metastasis inhibition complicates the study of the treatments’ effects. There are few models of *in vivo* relapse assays [45, 46]. Here we have used the second re-injection in re-xenograft model to asses this metastatic capacity. PEDF derived-peptides produce a decrease in cell resistance and percentage of resistance cell population after *in vivo* re-xenograft assays. Consequently, these re-xenografts from cells treated with PEDF-derived peptides showed statistically significant decreases of Tics markers (unpublished data). The most significant effect was observed after re-injection of treated xenograft cells. 70% of xenograft cells treated with CTE were not able to produce a new tumour (metastasis-like model). Regarding CT treatment, the effects were not so drastic, and we observed a 40% reduction in tumour growth. This is the first time that a treatment is able to modify the behaviour of cancer; modified initiating cells hindered the tumour′s re-growth, and therefore that of the metastasis animal model.

To sum up, this data suggests that PEDF-derived peptides play a potentially therapeutic role in colorectal cancer. In light of the findings reported here, it would be necessary to confirm the low toxicity and high efficacy of these peptides with additional pre-clinical and clinical trials. Moreover, it would be essential that these trials be carried out with purified CT/CTE peptides instead of conditioned medium. Nonetheless, preliminary studies indicate a similar effect in all of the cell lines when conditioned medium is replaced with purified peptides (data not shown). Furthermore, it would be interesting to evaluate the effect of PEDF-derived peptides on other types of cancer and also perform combinatorial analyses for a larger ratio of doses. The group is currently focused on elucidating the molecular mechanisms that underlay these observed effects. Tumour initiating cells are long-retaining labeling cells with asymmetric divisions [47]. We postulate that the PEDF signaling pathway is implicated in the up-down regulation of this asymmetric versus symmetric division. This could be one of the reasons why tumour initating cells do not disappear from the tissues after treatments. The PEDF protein has been related to several transmembrane receptors [30, 31], and another important challenge is the identification of the receptor involved in this process. The phosphorylation stage and the charge distribution of the amino acids are factors involved in the receptor-ligand binding [48], and in our case, the negative charge included in CTE could explain the robust effect observed with this molecule. Whether or not our hypothesis of asymmetric division regulation by the PEDF signaling pathway is finally checked, the potential risks of the clinical application of this molecule come from systemic application, because it would affect normal stem cells in regenerating tissues. However, there are possible strategies to avoid systemic treatment, which should be studied in this case. One way with great potential for development of drug delivery systems is molecule redirection by magneto-mechanical actuation.

## MATERIALS AND METHODS

### Cell lines and cell culture

The human colon cancer cells DLD-1, HT-29, SW-480 and SW-620 were obtained from Mario Fraga′s laboratory and maintained in DMEN F12 (Lonza Waldersville, Inc) supplemented with 10% fetal bovine serum (FBS, Lonza Waldersville, Inc), 200 nM L-glutamine (Lonza Waldersville, Inc) and 1% penicillin/streptomycin (Biowhittaker) in an atmosphere of 5% CO_2_ at 37° C in a humidified incubator.

### Animals

FOX^n^1nu, at 1 month of age and of indistinct gender were obtained from Charles River International Laboratories. Animals were housed and bred at 20–25° C, humidity of 50–60% and a 12 hours light-dark cycle. All of the animals were treated in accordance with the approval of the local ethics committee (University of Castilla–La Mancha).

### Pedf c-terminal conditioned medium

C-terminal (CT) fragment of PEDF protein was cloned in the mammalian expression vector pcDNA 3.1 (-)-myc-His as we describe previously [49]. The recombinant molecule was transiently transfected in HEK293T cells using conventional calcium phosphate protocol [50]. Cells were cultured up to a total of 5 days at 30° C. Afterwards the culture medium containing the secreted recombinant protein was harvested and was used in the following experiments as conditioned medium for cancer cells. Transfected cells with the empty vector were used as control medium to add to the control assays in the same proportion. The same procedure was carried out in the case of the CTE fragment, excepting a previous step of side directed mutagenesis so as to replace the serine residue of cCterminal PEDF to a glutamic residue with the intention of inhibiting the C-terminal phosphorylation site. Recombinant CT/CTE peptides in the medium were detected and quantified by western blot using known concentrations of purified PEDF protein. PEDF was purified by affinity chromatography using a polyhistidine tag. A calibration curve was elaborated to determine the exact CT/CTE peptides concentration to be applied in the experiments (Supplementary Figure 1). Western blot analyses were carried out with a primary antibody anti-c-Myc (Mouse monoclonal IgG1, Santa Cruz) in a 1:500 dilution overnight. The secondary antibody was a goat anti-mouse IgG HKP (Santa Cruz).

### C-terminal pedf peptides treatment

Colon cancer cell lines were treated with PEDF derived peptides (CT or CTE) at a final concentration of 8 nM (200 ng/ml) in the medium. Cells were treated in two ways. On the one hand, acute treatments were performed, that is, a single treatment with the peptides. On the other hand, chronic treatments were carried out consisting of a total of 6 treatments with the peptides for a total of fourteen days. Peptide treatment was added with culture medium in the passage time. Timing for the treatments was: two hours with 8 nM peptide exposition before chemotherapy treatment for acute treatments and six weeks with 8 nM peptide added to the culture medium for chronic ones.

### Crystal violet assay

5,000 cells were seeded per well in 24-well plates, in a volume of 250 uL. The next day the cells were treated with increasing doses of chemotherapeutic agents and left in a humidified incubator at 37° C and 5% CO2 for 4 days. Subsequently the cells were fixed with 0.5% glutaraldehyde (Sigma) for 10 minutes. Next, the cells were stained with 0.1% crystal violet for 20 minutes. After several washes, the staining was solubilized with 10% acetic acid. Finally, a spectrophotometric reading was performed at an onsa length of 590 nm with a plate spectrophotometer (Epoch, Biotek). The IC50 was determined (the dose of drug necessary to eliminate 50% of the cell population), while the IC0 (the dose of drug necessary to eliminate 100% of the population) was obtained through logarithmic regressions made with the program DE.0 plus v1.0.

### Xenografts

Untreated cells (control) and cells treated with peptides derived from PEDF suspended in PBS were injected subcutaneously on both flanks of immunocompromised mice. The injections were of 5000 or 500 cells, in a final volume of 200 ul of a 1: 1 dilution of matrigel (BD Matrigel^™^ Basement Membrane Matrix, BD) with a 25 gauge-needle. The growth of the tumours was monitored weekly by means of a caliper. The final tumour volume was calculated using the formula V= 2 × L_1_ × L_2_ × π/6. Tumours were mechanically and chemically disintegrated with EDTA at 37° C. Later they were washed with PBS and seeded in DMEN F12 medium (Lonza Waldersville, Inc) supplemented with 200 nM L-glutamine (Lonza Waldersville, Inc), 1% penicillin/streptomycin (Biowhittaker), 0,5% EGF and 0,04% FGF in an atmosphere of 5% CO_2_ at 37° C in a humidified incubator.

### Software and statistical analysis

To analyze the interaction between molecules we have used the software Drug Combination Computer Simulation CompuSyn (http://www.combosyn.com/) from Combosyn Inc. company. Also we have analyzed the results with the Combenefit software, also indicated to analyze synergy between molecules (https://www.cruk.cam.ac.uk/research-groups/jodrell-group/combenefit).

The statistical analysis was carried out using a Mann–whitney *U* test or the Chi-square test when necessary. The data is expressed as the mean plus the standard error (+SE). The obtained results are considered statistically significant when *p* < 0.05(^*^), *p* < 0.01(^**^) and *p* < 0.005(^***^).

## SUPPLEMENTARY MATERIALS FIGURES



## Abbreviations

IC50: concentration of drug necessary to kill 50% of the cell population
CT: carboxi-terminal part of the PEDF protein
CTE: carboxi-terminal part of the PEDF protein with a change of a serine by a glutamic
PEDF: Pigmented epithelium derived factor
