# Supplementary material for: Pedf derived peptides affect colorectal cancer cell lines resistance and tumour re-growth capacity
Source: Oncotarget. 2019 Apr 26;10(31):2973–86. doi: 10.18632/oncotarget.26085 (PMC6508205; doi:10.18632/oncotarget.26085)
Supplement: Supplementary file 1 [file oncotarget-10-2973-s001.pdf]

Pedf derived peptides affect colorectal cancer cell lines resistance and tumour re-growth capacity

SUPPLEMENTARY MATERIALS

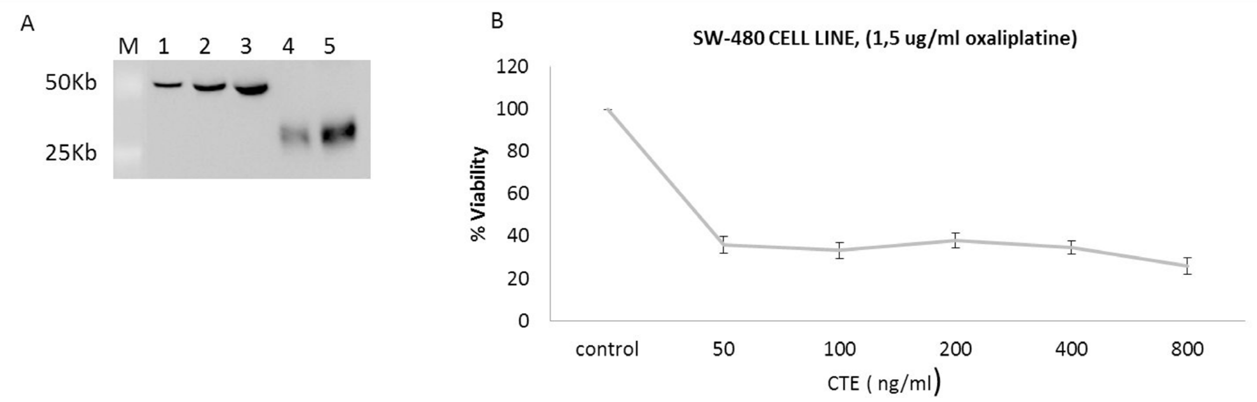

Supplementary Figure 1: (**A**) Western blot showing the PEDF curve used for quantification with 2 µl (line 1), 4 µl (line 2) and 6 µl (line 3) of 100 ng/µl PEDF. Line 4 corresponds to 15 µl of Cter peptide in the conditioned medium and 30 ul in line 2. (**B**) Changes in viability and doses-response curve behaviour in SW-480 colorectal cancer cell line with oxaliplatine treatment combined CTE peptide increasing concentration in acute treatment. Figure shows dose-response curve of oxaliplatin chemotherapeutic combined with CTE treatment conditions. An asymptotic effect is reached from 50 ng/ml to 800 ng/ml peptide concentration. 200 ng/ml concentration was selected to use as a range of action, but without necessarily reaching the maximums tested. Data represented as mean ± SEM.

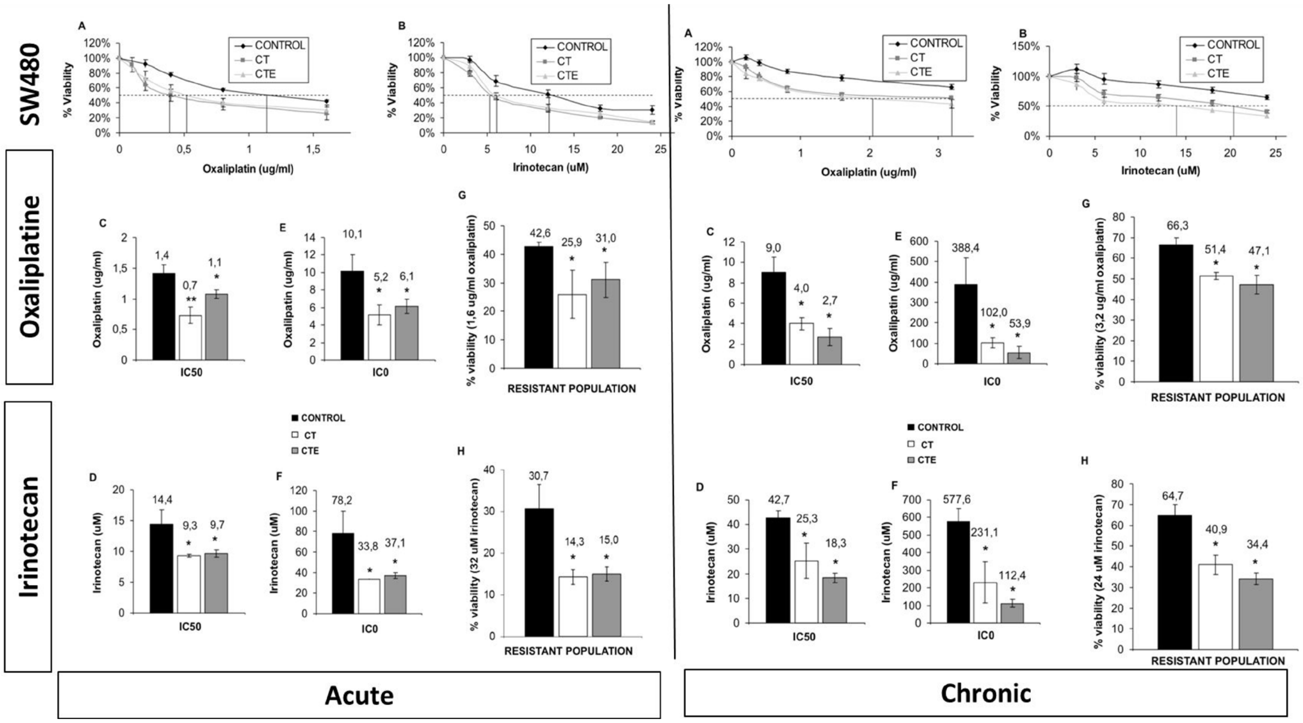

Supplementary Figure 2: Changes in IC50 and doses-response curve behaviour in SW-480 colorectal cancer cell line with oxaliplatine and irinotecan treatments combined CT and CTE peptides in acute and chronic treatment. Figure shows dose-response curve of both chemotherapeutics, oxaliplatine (**A**, **C**, **E** and **G**) and irinotecan (**B**, **D**, **F** and **H**) in the different CT or CTE treatments conditions: acute (left panels) and chronic (right panels). IC50 (C and D), IC0 (E and F) and resistant population (G and H) grafts, for every condition. Control bars (in black) use data from cells with chemotherapy but not CT (white bars) or CTE (gray bars) treatment. Data represented as mean ± SEM ($^{*}p > 0.05$, $^{**}p > 0.01$, $^{***}p > 0.001$).

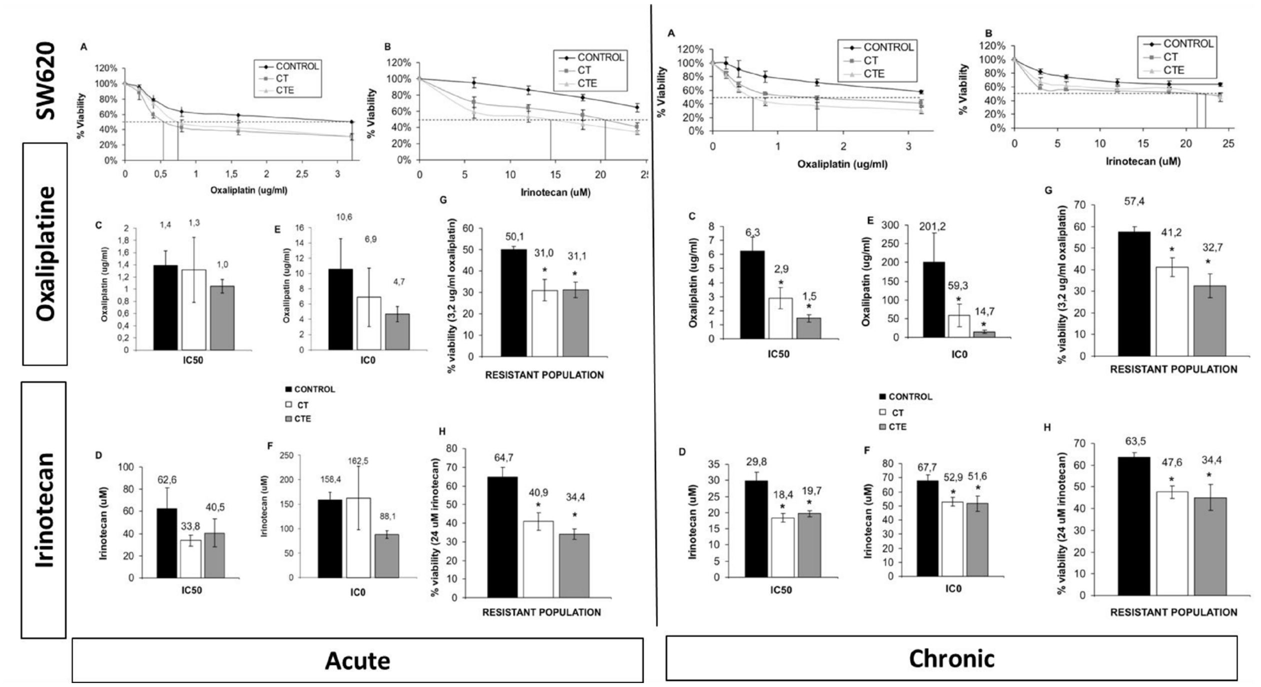

Supplementary Figure 3: Changes in IC50 and dose-response curve behaviour in SW-620 colorectal cancer cell line with oxaliplatine and irinotecan treatments combined CT and CTE peptides in acute and chronic treatment. Figure shows dose-response curve of both chemotherapeutics, oxaliplatine (**A**, **C**, **E** and **G**) and irinotecan (**B**, **D**, **F** and **H**) in the different CT or CTE treatments conditions: acute (left panels) and chronic (right panels). IC50 (C and D), IC0 (E and F) and resistant population (G and H) grafts, for every condition. Control bars (in black) use data from cells with chemotherapy but not CT (white bars) or CTE (gray bars) treatment. Data represented as mean ± SEM ($^{*}p > 0.05$, $^{**}p > 0.01$, $^{***}p > 0.001$).

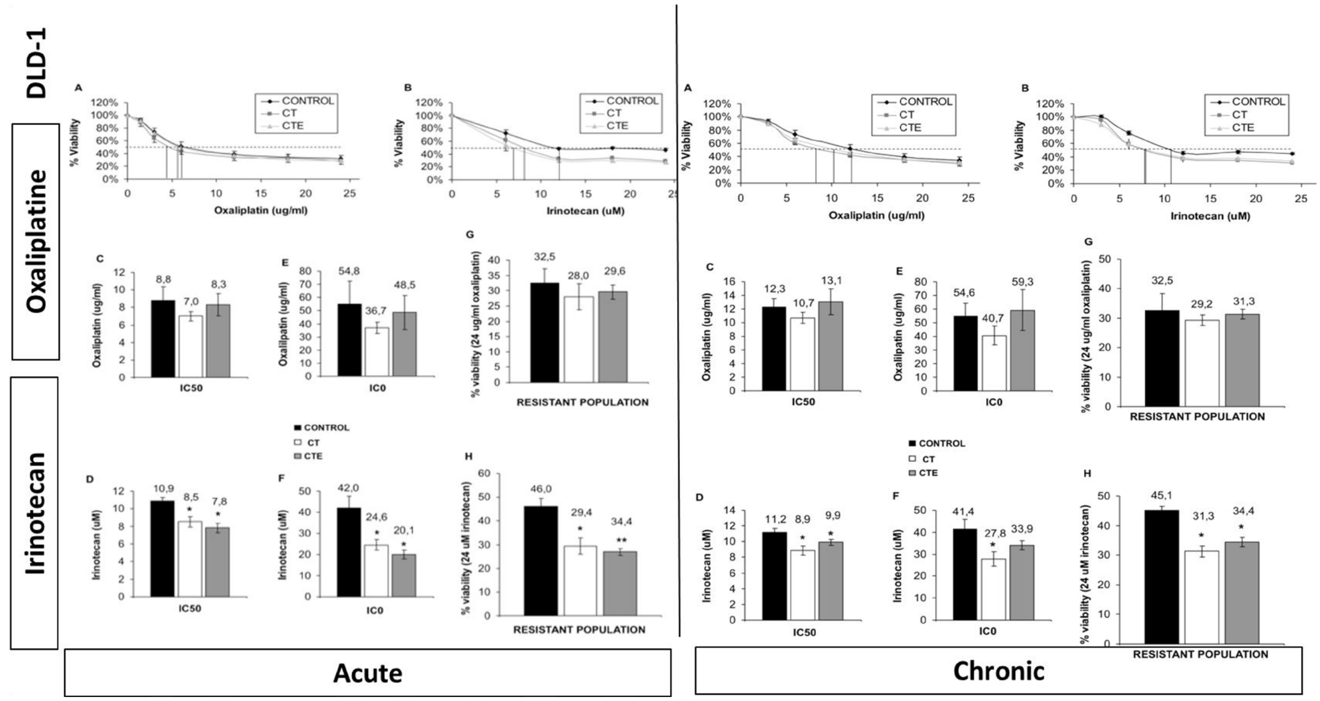

Supplementary Figure 4: Changes in IC50 and dose-response curve behaviour in DLD-1 colorectal cancer cell line with oxaliplatine and irinotecan treatments combined CT and CTE peptides in acute and chronic treatment. Figure shows dose-response curve of both chemotherapeutics, oxaliplatine (**A**, **C**, **E** and **G**) and irinotecan (**B**, **D**, **F** and **H**) in the different CT or CTE treatments conditions: acute (left panels) and chronic (right panels). IC50 (C and D), IC0 (E and F) and resistant population (G and H) grafts, for every condition. Control bars (in black) use data from cells with chemotherapy but not CT (white bars) or CTE (gray bars) treatment. Data represented as mean ± SEM ($^{*}p > 0.05$, $^{**}p > 0.01$, $^{***}p > 0.001$).

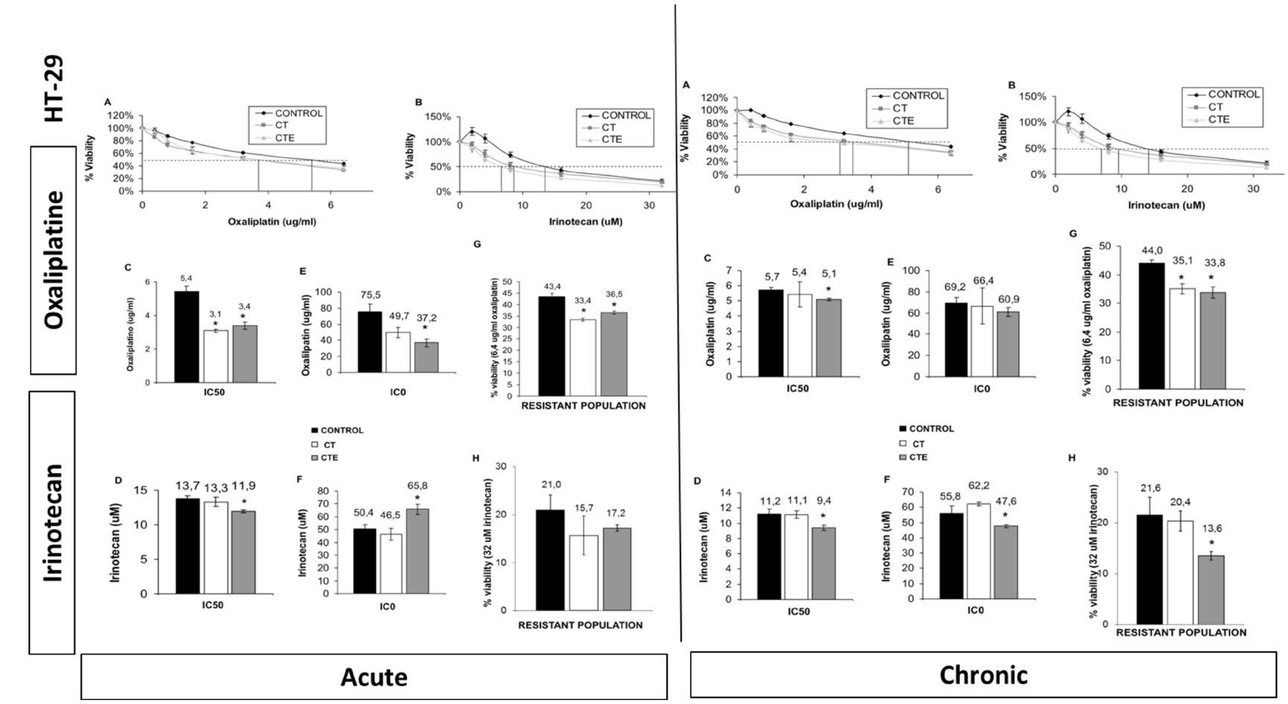

Supplementary Figure 5: Changes in IC50 and dose-response curve behaviour in HT-29 colorectal cancer cell line with oxaliplatine and irinotecan treatments combined CT and CTE peptides in acute and chronic treatment. Figure shows dose-response curve of both chemotherapeutics, oxaliplatine (**A**, **C**, **E** and **G**) and irinotecan (**B**, **D**, **F** and **H**) in the different CT or CTE treatments conditions: acute (left panels) and chronic (right panels). IC50 (C and D), IC0 (E and F) and resistant population (G and H) grafts, for every condition. Control bars (in black) use data from cells with chemotherapy but not CT (white bars) or CTE (gray bars) treatment. Data represented as mean ± SEM ($^{*}p > 0.05$, $^{**}p > 0.01$, $^{***}p > 0.001$).
